# Generating Synthetic Labeled Data From Existing Anatomical Models: An Example With Echocardiography Segmentation

**DOI:** 10.1109/TMI.2021.3051806

**Published:** 2021-01-14

**Authors:** Andrew Gilbert, Maciej Marciniak, Cristobal Rodero, Pablo Lamata, Eigil Samset, Kristin Mcleod

**Affiliations:** 1 GE Vingmed Ultrasound, GE Healthcare 3183 Horten Norway; 2 Department of InformaticsUniversity of Oslo 6305 0315 Oslo Norway; 3 Biomedical Engineering DepartmentKing’s College London 4616 London WC2R 2LS U.K.

**Keywords:** Data generation, echocardiography, generative adversarial networks, segmentation, synthesis

## Abstract

Deep learning can bring time savings and increased reproducibility to medical image analysis. However, acquiring training data is challenging due to the time-intensive nature of labeling and high inter-observer variability in annotations. Rather than labeling images, in this work we propose an alternative pipeline where images are generated from existing high-quality annotations using generative adversarial networks (GANs). Annotations are derived automatically from previously built anatomical models and are transformed into realistic synthetic ultrasound images with paired labels using a CycleGAN. We demonstrate the pipeline by generating synthetic 2D echocardiography images to compare with existing deep learning ultrasound segmentation datasets. A convolutional neural network is trained to segment the left ventricle and left atrium using only synthetic images. Networks trained with synthetic images were extensively tested on four different unseen datasets of real images with median Dice scores of 91, 90, 88, and 87 for left ventricle segmentation. These results match or are better than inter-observer results measured on real ultrasound datasets and are comparable to a network trained on a separate set of real images. Results demonstrate the images produced can effectively be used in place of real data for training. The proposed pipeline opens the door for automatic generation of training data for many tasks in medical imaging as the same process can be applied to other segmentation or landmark detection tasks in any modality. The source code and anatomical models are available to other researchers.^1^^1^https://adgilbert.github.io/data-generation/

https://adgilbert.github.io/data-generation/

## I. Introduction

Medical imaging provides a window to capture the structure and function of internal anatomies. Imaging modalities such as ultrasound, computed tomography (CT) or magnetic resonance imaging (MRI) can be used to measure physical and physiological parameters. Accurate automation of these measurements would provide significant time-savings for clinical practitioners.

Convolutional neural networks (CNNs), have become the candidates of choice for measurement automation because they can accurately learn complex relevant features. However, CNNs require large sets of labeled data to learn and they are limited in accuracy by the quality of labels used in training. Inter-observer errors can be high in medical imaging tasks, especially when there is noise or other artifacts in the image. For example, in cardiovascular ultrasound (echocardiography or ‘echo’), inter-observer errors for labeling common measurements can range from 4-22% even for experienced cardiologists [1], [2]. The variability in measurements is due to (a) the difficulty of accurately interpreting signals delineating structures amid image noise, and (b) differences in implementation between different acquisition machines and between practitioners in different institutions. A second problem when building datasets to automate tasks in medical imaging is labeling is time-consuming and expensive since quality annotations require experienced medical professionals. Finally, manual labels are inflexible and adapting them based on new insights requires a significant amount of time.

**Fig. 1.**
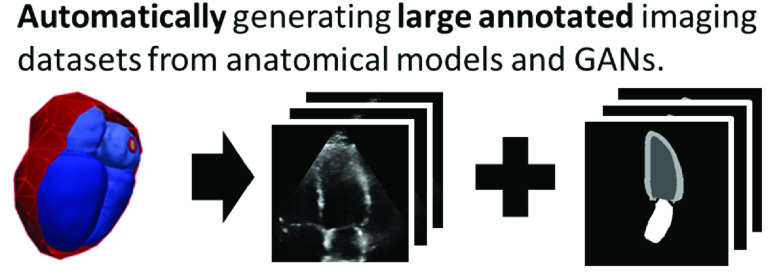
Using anatomical models as high quality ground truth annotations, we propose a pipeline to generate large synthetic datasets for training convolutional neural networks.

While CNNs have been at the forefront of automating diagnostic measurements, anatomical models are progressing the personalization of treatments. Simulations from “digital twins” (models with patient-specific parameters) are increasingly being used to guide therapies and develop new treatments [3]. As with the revolution in statistical inferencing led by deep learning, larger computational resources have allowed the growth in complexity and realism of these anatomical models [4], [5]. While originally developed for personalized simulation of mechanics and biophysics, anatomical models are also a valuable source of high-quality shape information. We propose a method to solve the labeling challenges for medical deep learning by harnessing the information contained in anatomical models. Instead of labeling images, we let these models represent ground-truth anatomical shapes and generate task-specific paired realistic images as summarized in Fig. 1.

In particular, we demonstrate the usefulness of this pipeline for the task of segmenting parts of the left heart in echo images and thus make use of a set of cardiac models developed for electromechanical simulations of the heart. Similar anatomical models have been developed for a wide range of anatomies and most are free for academic use [4], [6]. The pipeline described here could readily be applied to those models as well with some application specific modifications. Section V-E provides more details on extensions to new anatomies.

### A. Contributions

The proposed pipeline shifts the focus from annotating images to ensuring a CNN trained on synthetic images will generalize to real images. We test our pipeline by generating synthetic data for echo segmentation. Our main contributions are three-fold:

1) We present a pipeline to generate realistic synthetic images with paired labels using anatomical models and a CycleGAN [7]. The pipeline can generate datasets of arbitrary size and include labels from any region included in the original anatomical models.
2) We demonstrate the utility of the pipeline by building annotated synthetic 2D echo images from cardiac models. We show these synthetic images can be used for training deep learning algorithms, specifically by demonstrating accurate segmentation without any real labeled images. We present extensive validation of the proposed pipeline by testing on multiple datasets of real images from different clinical sites and annotators that were completely unseen during development.
3) We present an analysis of the sources of error in the segmentation including differences in image texture, tissue shape, and annotator style. We show that differences in the segmentations primarily come from differences in annotator bias, highlighting the need for standardized annotations.

### B. Related Work

Because there are often only a few accurate anatomical models available, we first experiment with using shape analysis techniques to expand the available set of ground truth models. Shape analysis has previously been used in medical imaging for improving segmentations as well as for pathology detection and registration [8]–[11].

The proposed work translates labels from a source domain (slices from anatomical models) to a target domain (echo in the example application). Domain adaptation is a similar task, but uses labels from a different imaging modality instead of models. Recently, CycleGANs have facilitated domain adaptation with unpaired images by using two sets of generative and discriminative networks, one for each transformation direction [7]. Kazeminia *et al.* [12] and Taghanaki *et al.* [13] provide overviews of CycleGANs in medical imaging. So far CycleGANs have primarily been used for realistic cross-modality translation to CT or MRI images whereas this work focuses on echo. Generating echo images is challenging because of the complex noise patterns. These patterns change dramatically between images and even within a single image following the acquisition settings of the user and the stretching/squeezing of the scan-conversion process. Compared to echo, the well-defined boundaries in MRI or CT represent a more similar domain to the anatomical model images. The cone in echo images is also a consistent defining feature in the image which degrades the translational invariance of convolutional networks. CycleGANs have been applied in echo for segmentation with image quality improvement [14] and view conversion [15], but these works used two real datasets of echo images and thus did not have to address the above challenges of translating from a different modality to echo.

Others have developed alternative strategies for surmounting limited datasets in medical imaging and Tajbakhsh *et al.* provide an overview of different strategies for segmentation with unlabeled or limited data [16]. Specifically relevant to this work, several groups have proposed strategies using GANs to generate synthetic data. Eschweiler *et al.* proposed a CycleGAN strategy for synthesizing a microscopy cell image and location dataset [17]. However, their labels are randomly generated, which loses the key advantage of ground truth anatomical models and is not applicable to most other applications in medical imaging where anatomies cannot be randomly generated from scratch. Huo *et al.* proposed SynSeg-Net, a similar pipeline using unpaired labels from MRI to train networks on CT images using a CycleGAN [18]. While some of the methodologies are similar, the central difference is that our ground truth annotations come from 3D anatomical models rather than unpaired images from another modality. Because detailed 3D annotations are an intrinsic part of each anatomical model, *our pipeline is applicable to any segmentation or landmark detection task in any modality with no additional labeling required*. Our approach is focused on image synthesis rather than domain adaptation.

Previous works generating echocardiography images have primarily used physics simulators to exactly replicate speckle creation from a set of reflectors. In general, these approaches have focused on generating a few specific images rather than large datasets. For example, Alessandrini *et al.* demonstrated a full pipeline for generation of 3D echo video loops that were realistic enough to trick some human observers [19]. While useful for providing a ground truth of myocardial motion for strain estimation, this pipeline and similar approaches [20]–[22], are ill-suited for generating training data for deep learning algorithms because it does not scale well to larger datasets. Each new generated image requires manual initialization and computationally heavy simulation. Other groups have used generative adversarial networks (GANs) for echo image synthesis. For example, Abdi *et al.* sampled new echo images from labels after conditioning a GAN on a paired dataset [23] an approach also demonstrated for skin lesions [24]. The key drawback is this approach can only be used to augment existing, already annotated datasets.

## II. Methods

**Fig. 2.**
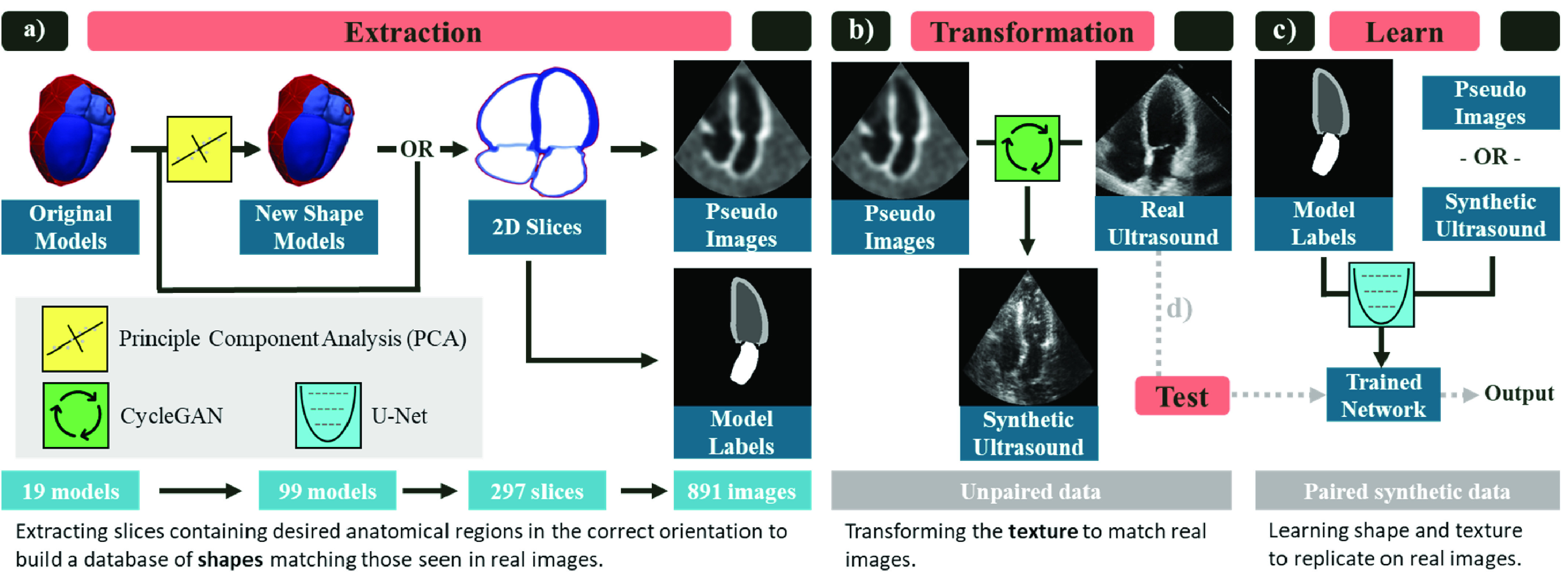
**Overview of proposed pipeline implemented for echocardiography segmentation.** a) Extraction: pseudo images and ground truth labels are built from the 3D anatomical models. First, a larger cohort of shapes is generated by building a statistical shape model from the original anatomies and sampling new 3D instances using principle component analysis (PCA). Next, 2D slices of the desired view (apical four chamber shown) are sampled. Finally, pseudo-ultrasound images and the corresponding labels are built. Each step expands the size of the dataset. b) Transformation: The pseudo images and a dataset of unlabeled real echo images are used to train a CycleGAN to transform the pseudo images into synthetic ultrasound images. c) Learn: The paired synthetic ultrasound images and model labels are used to train a U-Net segmentation network. d) Test: The network trained on synthetic images is tested on real images to evaluate the utility of the pipeline. The creation of new shape models as well as the transformation module are optional extensions. The slicing can be performed on the original models and the segmentation network can be trained using pseudo images instead. We evaluate the effectiveness of these components in Sec. IV.

The proposed pipeline consists of two primary steps as shown in Fig. 2. First, pseudo images and paired labels are generated from 3D anatomical models as described in Sec. II-A. Second, the pseudo images are transformed into realistic synthetic ultrasound images using a CycleGAN and a set of real images as described in Sec. II-B. Afterwards, in Sec. II-C, we test the utility of the generated datasets by comparing segmentation networks trained with synthetic images to those trained with real images when testing on real images. The proposed pipeline is general to any medical imaging application, although models for the relevant anatomy are needed and application specific parameters are required in the extraction. Sec. III-A describes the models used for this application and Sec. III-B provides details on the example application (echo segmentation) and parameters. We demonstrate the method using several datasets as described in Sec. III-D.

### A. Extraction

The input to the pipeline is a small set of anatomical models (“Original Models”) which contain labels for the attributes that will be segmented or detected.

#### 1) New Shape Models:

While anatomical models provide excellent ground truth, there are often few available which may not provide sufficient anatomical variability to build a heterogeneous dataset. We experiment with building additional anatomically realistic models using statistical shape analysis. The primary modes of variation are deconstructed from the original models using principle component analysis. New models are generated by randomly sampling from the first 9 modes of variation (capturing 90% of the total variation) within two standard deviations of the mean model. We repeat this procedure to generate 99 new models in total. Each of the synthetic models is still an anatomically plausible shape, but adds a heterogeneous example to our dataset. Full details of the construction are given in Appendix A.

#### 2) Pseudo Images and Model Labels:

A CycleGAN learns to transform the appearance from one imaging set to another. To generate the input for the CycleGAN, a “pseudo” database is generated where the anatomical shapes present in the pseudo database generally match the shape distribution found in an equivalent database of real images. Therefore the necessary functions here are application specific and are discussed in detail in Sec. III-B. The output of this step is both a pseudo image and a label image which contains ground truth for the learning step. Synthetic labels are generated from the original model to match the chosen task and selecting the task simply involves choosing the relevant regions in the model.

### B. Transform

A CycleGAN [7] is trained to transform the pseudo images into synthetic ultrasound images using an unlabeled set of real ultrasound images. The default CycleGAN architecture and hyper-parameters are used except the generator network is replaced with a U-Net with 8 down-sampling levels [25] because it trains faster and gives equivalent results. The CycleGAN is trained for 200 epochs. Network weights are saved every 5 epochs. We select the best epoch by manually reviewing a sample result from each epoch (typically around epoch 180) but the exact epoch chosen did not have a significant impact on results in preliminary experiments. The selected network is used to save a synthetic image and paired label for each pseudo image.

### C. Learn

A segmentation network is trained from the set of generated synthetic images and labels. The same U-Net architecture from the transform step is used. The network is trained for 30 epochs using cross-entropy loss. While the segmentation network can be included within the CycleGAN for end-to-end training [18], [26], we found the segmentation network was able to consistently achieve very good results on the synthetic images in preliminary results and did not find value in including this as a loss term within the transformation process. Additionally, splitting these two steps allowed us to develop an equal comparison between the synthetic and real data.

## III. Example Application: Echo Segmentation

**Fig. 3.**
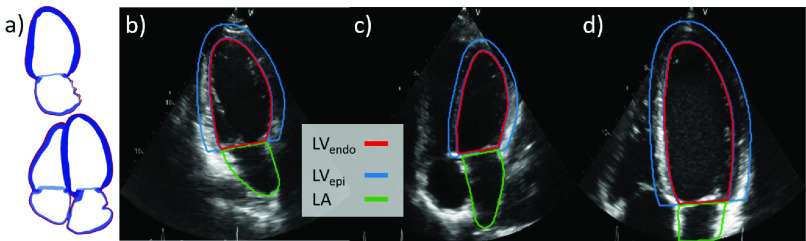
**Example application: echo segmentation.** a) Apical two chamber (top) and apical four chamber (bottom) views as shown in an anatomical model. The right images show example real apical two chamber (b) and apical four chamber (c, d) echo images with task labels. (b, c) show the full heart while d) is zoomed to focus on the left ventricle.

The feasibility of the pipeline is proven by building synthetic datasets for 2D echo segmentation. This application was chosen to enable comparison against existing real datasets. Two task variants are tested. First, matching all overlapping constraints of the synthetic and real datasets presented in Sec. III-D, a network was trained to segment the left ventricle endocardial border (LV_endo_) from apical four chamber images taken from the end diastole phase of the cardiac cycle. Second, the task was extended to include the left ventricle epicardial border (LV_epi_) and left atrium (LA) border from both four chamber and apical two chamber views and both end diastole and end systole phases. Fig. 3 shows examples of apical four/two chamber images extracted from the anatomical model as well as examples of performing the relevant annotations in real ultrasound images.

### A. Original Models

The original models for this application were a set of 19 3D heart models derived semi-automatically from CT images. CT images have high contrast and spatial resolution which enables accurate delineations of structure boundaries. These models were built for electromechanical simulations and contain a complete set of tissue labels. Each model contains labels for both ventricles, both atria, aorta, pulmonary artery and veins, and both venae cavae. Additional details on the model creation process are given in Appendix A and by Rodero *et al.* [27] (currently under review, the model construction matches that described in [28]).

### B. Task-Specific Data Generation

To generate a dataset for this task, 2D slices were sampled from the anatomical models and masked to mimic ultrasound images. A perfect 2D apical four chamber image is defined as the plane intersecting the apex, mitral valve center, and aortic valve center [29]. These landmarks were extracted from each model to define the optimal plane. Apical two chamber images were extracted by performing a 70 degree rotation counter-clockwise around the apical long axis from the four chamber landmarks (see Fig. 3). Although clinical guidelines suggest rotating the probe 60 degrees [29], using 70 degrees gave a better cut plane for the models from qualitative evaluation. To mimic natural variation in acquisition, random rotations of the cut planes around the long and short axes of the LV were sampled so that some slices are foreshortened or off-plane.

The 2D slices were transformed into pseudo images which mimic the appearance of ultrasound images. One of the most distinguishing features of an ultrasound image is the ‘cone’ marking the boundaries of imaging data. This is a consistent strong feature in all images and we found that the translational invariance of CNNs is degraded because the network could learn relationships between the cone boundary and structures. In other words, the CycleGAN discriminators could find difference between real and synthetic images from differences in structure location. In response, the generators would hallucinate structures in random locations. For the CycleGAN to properly transform structures as well as appearance, it is important that the distributions of locations of different anatomical structures are equally represented in both datasets.

**Fig. 4.**
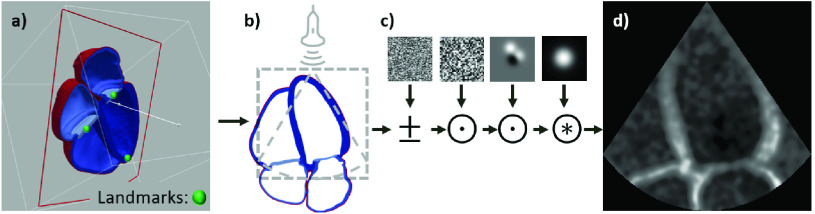
**Extraction details for echocardiography.** To extract pseudo images a) a 2D plane is defined from a set of landmarks, b) the plane is rotated and cropped to match standard acquisition parameters and positioned to match standard positioning in real images, c) random noise and shadows are added and the slice is blurred yielding d) the final pseudo image. Additional details are given in Appendix A.

To match this constraint, a series of affine transformations were applied to mimic the different LV orientations found in real images. This primarily consisted of masking the image with a cone and randomly cropping to either the entire heart (‘whole heart’ image) or the LV (‘LV focused’ image). The different crops are shown in Fig. 3 and match the image types suggested in clinical guidelines [29]. After cropping, other affine transforms such as rotations and squeezing were applied to ensure the region of interest remains inside the cone and add variance to the dataset (see Appendix A). Hard edges also decreased the realism of the generated images (see Appendix F) so random uniform noise and shadowing was added and the images were blurred by convolving with a Gaussian kernel. This process is shown in Fig. 4. To introduce additional variety, the slicing and pseudo extraction processes were repeated 3 times each for a total of 891 images (99 $\times $ 3 $\times $ 3). The entire process is fully automated.

### C. Segmentation Evaluation and Network Selection

Several metrics were used to evaluate the accuracy of the trained segmentation networks. First, the Dice score was measured where $\boldsymbol {D} = 200*(S_{pred} \cap S_{ref})/(S_{pred} + S_{ref})$ and measures the overlap between a predicted segmentation $S_{pred}$ and a reference segmentation $S_{ref}$. Second, following [30], we analyzed the convexity and simplicity of the output as criteria which identify successful annotations. Because we found these two metrics vary together, only simplicity is reported. Simplicity is defined as $\boldsymbol {S_{p}} = \sqrt {4*\pi *Area(S_{pred})}/Perimeter(S_{pred})$ [31]. Note that simplicity relies only on the segmentation mask output from the network $S_{pred}$ and not the label mask $S_{ref}$.

For the task of LV_endo_ segmentation, differences between annotators are often because of differing placements of the endocardial border within the myocardial tissue rather than differing ventricular shapes. According to guidelines [29], the LV_endo_ border falls at the interface between the non-compacted and compacted myocardium. If this border cannot be determined then the border falls at the blood-tissue interface. In noisy ultrasound images it can be difficult to accurately label this border, and there may be disagreement about which criteria should be used. There are no clear guidelines established for labeling LV_epi_ and LA borders for segmentation [30] which can lead to differences between annotators for those tasks as well.

To capture these potential disagreements, we calculated several additional metrics: mean distance between the contours and Bias. Mean distance ($d_{m}$) is the distance between two contours $C_{ref}$ and $C_{pred}$ averaged across their length. $C_{x}$ indicates the border of $S_{x}$. Bias is the percentage error between the segmentation areas and is defined as:

\begin{equation*} \boldsymbol {B} = 200*\frac {Area(S_{pred}) - Area(S_{ref})}{Area(S_{pred}) + Area(S_{ref})} \tag{1}\end{equation*}

A high average Bias (positive or negative) across a dataset indicates a systematic difference in the labeling since the predicted results are consistently larger/smaller than the reference. Mean distance is calculated in pixels since we do not have access to image sizes in mm for all datasets. All other metrics are unit-less.

All networks were able to achieve high Dice scores on the synthetic data in preliminary experiments so selecting the network based on best Dice on a synthetic validation set lead to over-fitting to the synthetic data. Simplicity is a marker of the annotation quality that relies only on the network output and does not require a label. Therefore simplicity was tracked on an unlabeled set of real images (separate from the test set) through the course of training and the network with the highest simplicity was selected for final testing. This choice encouraged networks that generalized well to real data without requiring labels.

Median metrics were calculated in all cases since the distribution of scores was not normal. Therefore median absolute deviation was used as a measure of variance where $\boldsymbol {MAD} = Median(\vert X_{i} - \tilde {X}\vert)$. The Wilcoxon signed-rank test was used to calculate statistical significance between different results [32].

### D. Real Datasets

Validating a dataset on a single source can lead to implicit bias in the developed methods [33]. For example, Degel *et al.* showed a decrease from 0.75 to 0.10 in Dice score for a CNN trained on one machine and tested on another for 3D left atrial segmentation. To account for this we validated the pipeline using a selection of real datasets. The characteristics of each dataset are described below and full details are listed in Appendix A.

#### 1) Camus:

The Camus dataset was introduced by Leclerc *et al.* [30]. It consists of apical four and two chamber images with segmentation labels for LV_endo_, LV_epi_, and LA at end diastole and end systole time points in the cardiac cycle. The images also include quality labels, and following the authors we limit our analysis to images of good or medium quality, leaving 1,600 images. The images are divided into training, validation and test splits of 80%, 10%, and 10% respectively, keeping images from the same patient in the same split.

#### 2) EchoNet:

The EchoNet dataset was introduced by Ouyang *et al.* [34]. It consists of 10,024 apical four chamber video loops with LV_endo_ segmentation labels for end diastole and end systole The images were divided into training, validation and test splits of 80%, 10%, and 10% respectively, keeping images from the same patient in the same split.

#### 3) Additional Real Datasets:

Since EchoNet contains only LV_endo_ annotations in apical four chamber images, additional real images were labeled with a full set of annotations, views and cardiac phases. Mixed apical four and two chamber videos from two different clinical sites were annotated by two experienced cardiologists ($O_{1}$ and $O_{2}$). Both cardiologists use echo as a part of their daily practice. To annotate the images they used the whole loop to check myocardial movement to find the correct structures and annotated LV_endo_, LV_epi_, and LA labels at end diastole and end systole ensuring that the labels between phases matched. The datasets were split by institution, **Site **_A_ contains 336 images and was further divided into training and validation splits of 80% and 20% respectively. **Site **_B_ contains 229 images and was left exclusively as a test set. Site_A_ was labeled by $O_{1}$ and Site_B_ was labeled by $O_{2}$.

#### 4) Pathological Dataset:

The anatomical models were derived from asymptomatic patients and the aforementioned datasets contain no information on patient diagnosis. Therefore a set of pathological images was also gathered to test how well the networks trained on real and synthetic images would be able to adapt to pathological cases. 61 exams were gathered from patients diagnosed with severe functional mitral regurgitation, which is correlated with significant changes in LV shape [35], [36]. A severe diagnosis corresponds to a rating of 4 on a 4 point scale of severity. A random apical four chamber image was selected for each patient and $O_{2}$ labeled LV_endo_, LV_epi_, and LA areas at end diastole and end systole (yielding 122 images total) using the same criteria as above. All images were used exclusively for testing.

### E. Synthetic Datasets

Synthetic versions of the Camus, EchoNet, and Site_A_ datasets were generated using the pipeline in Fig. 2. No synthetic dataset was generated for the Site_B_ or Pathological datasets since both were used for testing. Extraction and transformation were performed individually for each dataset and separately for each view. We predicted that using separate CycleGANs for each dataset and view would enhance the quality of the generated views and would allow the learned image features to be specific to the relevant dataset/view. Although customization of datasets and views could likely be combined into a single transformation process (using for instance an additional conditional input to the network), the focus of this work was on evaluating the feasibility of the pipeline rather than optimizing the generation process for multiple views and datasets. In most use cases all available datasets could be combined, however they were left separate here for evaluation purposes. Since Site_A_ contained fewer images, the CycleGAN for that dataset was initialized from the final trained CycleGAN from the Camus dataset and it was trained for only 100 epochs. The models were built for only a single time step so the synthetic datasets contain only end diastole images.

To test the impact of the new shape models, a synthetic EchoNet dataset was created without using the additional models generated in the shape extension described in Sec. II-A.1. To maintain dataset size, the extraction part was modified to extract 5 2D slices per anatomical model and 9 pseudo images per slice (for a total of 855 images). This set is denoted with an * in the experiments in Sec. IV.

### F. Inter-Observer Study

To analyze label variability, an inter-observer study was conducted for a subset of each dataset. 20 random images were selected from the test set (or validation if no test set was created) for the Camus, EchoNet, Site_A_, Synthetic Camus, Synthetic EchoNet and Synthetic Site_A_ datasets. To minimize the possible sources of variability, and match the overlapping constraints of the datasets, only apical four chamber end diastole images were selected. $O_{1}$ and $O_{2}$ annotated all images (except only $O_{1}$ labeled the EchoNet sets) with LV_endo_, LV_epi_ and LA labels. The second round of labeling was conducted at least 2 months after the first round for Site_A_.

### G. Implementation Details

Hyperparameters for the segmentation such as the learning rate and loss function were tuned on the synthetic validation sets. All approaches were evaluated on the Camus validation set to ensure proper convergence and several different validation runs were run in the course of building the extraction and transformation steps. In general, the goal of this work was to evaluate the synthetic dataset construction using standard segmentation approaches rather than tuning an optimal segmentation network for the given application. The unlabeled EchoNet and Site_A_ validation datasets were used only for network selection (see II-C) so the labels and metrics for these sets were never seen (and thus cannot influence design choices). This allows us to detect implicit bias in the design choices or training datasets. The test sets (Camus, EchoNet, and Site_B_) were used only once during final testing for the results presented below. Additional details on implementation and hyperparameters can be found in the supplementary material.

## IV. Results

The pipeline is evaluated first in Sec. IV-A by comparing expert cardiologist’s annotations to those produced by the proposed pipeline. Next, since the aim of this pipeline is primarily to generate image/label pairs that are suitable for deep learning training, we check if a CNN can effectively learn from synthetic images in Sec. IV-B and compare to networks trained on real data. Finally, various versions of the synthetic dataset are analyzed in Sec. IV-C to determine which factors contributed to accurate segmentations.

### A. Generated Images and Annotations

**Fig. 5.**
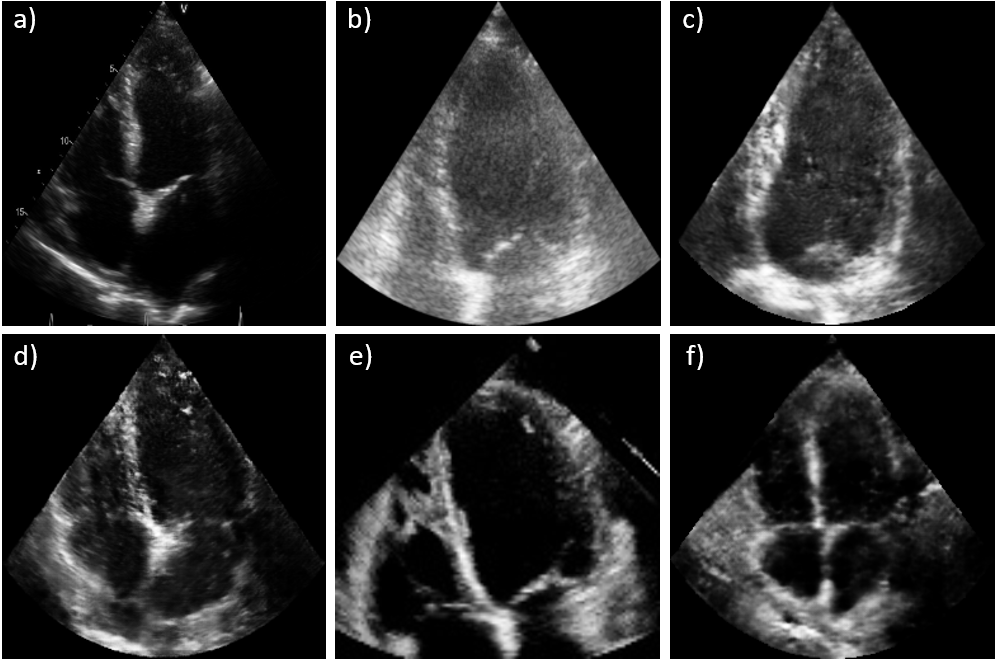
**Synthetic images closely match real images.** Can you guess which images are synthetic? Answers below.^3^

Images from the randomly selected inter-observer set are shown in Fig. 5 to demonstrate the realistic output of the generation pipeline. The synthetic images closely match their real counterparts in appearance. The GAN generates this appearance while maintaining the ground truth cardiac structures from the anatomical models. Generating a single ultrasound image from the prepared slice takes 81 ms.^3^a) Real Site_A_ b) Real Camus c) Synthetic Camus d) Synthetic Site_A_ e) Real EchoNet f) Synthetic EchoNet.

**Fig. 6.**
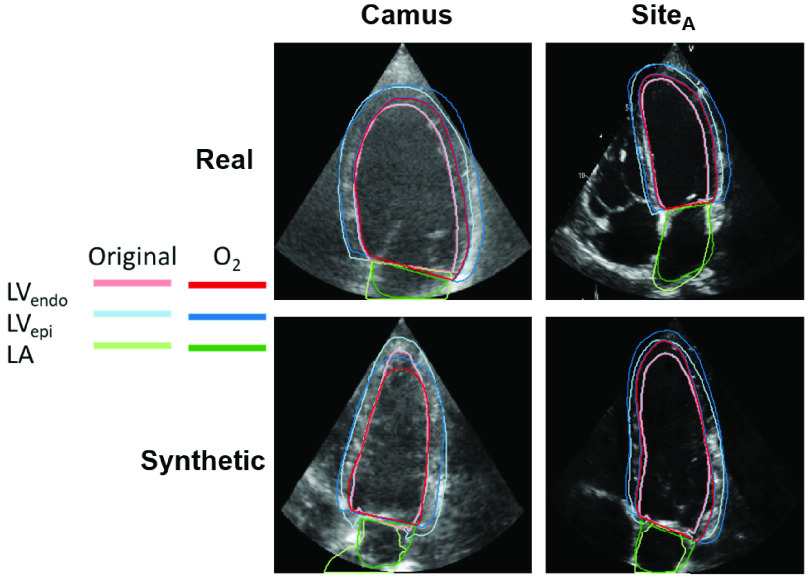
**Expert annotations on synthetic images match the anatomical model annotations at a level equal to inter-observer** error on real images: A sample image with included labels from the Camus and Site_A_ images used for the inter-observer study, chosen by taking the median Dice score between ${O}_{{2}}$ and the original labeler. For the real datasets the original labeler was [30] and ${O}_{{1}}$ for Camus and Site_A_ respectively. For the synthetic datasets the original label comes from the anatomical models.

Next, we checked if experts agreed with the pipeline-generated annotations. Metrics from the inter-observer study are shown at the top of Table I. $O_{2}$ had higher Dice scores on synthetic images than real images on LV_endo_ segmentations, was comparable for LV_epi_ and had higher scores on real images on LA segmentations. The median image in LV_endo_ Dice score between $O_{2}$ and the original annotator is shown in Fig. 6. Overall, $O_{2}$ closely matched the pipeline-generated labels although there was some disagreement in the apical region. Fig. 6 also shows that while structure consistency between pseudo and synthetic images was not explicitly forced in the CycleGAN, the synthetic structures remain true to the original annotation mask. Only the results from $O_{2}$ are used for comparison here for simplicity and because there was a large intra-observer bias in the results for $O_{1}$. The results from $O_{1}$ are presented in Appendix C and showed the same patterns as $O_{2}$ between synthetic and real. Finally, Fig. 5 and Fig. 6 shows the difference in appearance between the different datasets for both synthetic and real images. The Camus images are typically cloudier in appearance while the EchoNet/Site_A_ images usually have a higher gain setting and are thus clearer.

**TABLE I:** Synthetic Data Can Effectively be Used in Place of Real Data: Median Metrics Comparing Training With Real Datasets to Training With Synthetic Datasets. The First Section Compares Inter-Observer Results for ${O}_{{2}}$ on Real and Synthetic Data. The Next Section Shows Networks Trained on Real and Synthetic Data for LV_endo_ Segmentation in A4C ED Images and Tested on EchoNet. The Final Section Compares Networks Trained on Real and Synthetic Data for All Annotations/Views/Phases. All Dice Results Are Statistically Different With a p-Value < 0.05 (Computed With a Wilcoxon Signed-Rank Test) Except for the Marked Comparison (^†^). Results are Ordered by Dice Score and Bold Shows the Best Result in Each Section

Task	Training Data	Testing Data	$\boldsymbol {D}$ (%) [$\boldsymbol {MAD}$]			$\boldsymbol {B}$ (%)			$\boldsymbol {S_{p}}$	$\boldsymbol {d_{m}}$
	OR inter-observer comparison		LV_endo_	LV_epi_	LA	LV_endo_	LV_epi_	LA	LV_endo_	
Inter-	$O_{2}$ vs. Camus/Site_A_		87.9 [4.4]	93.4 [2.2]	87.5 [6.1]	24	8	−3	0.83	5.0
Observer	$O_{2}$ vs. proposed pipeline		90.8 [2.3]	92.0 [2.1]	83.0 [8.0]	9	4	14	0.80	3.9
LV_endo_ in A4C ED	EchoNet (base)	EchoNet	94.0 [1.6]	n/a	n/a	0	n/a	n/a	0.85	2.7
		Camus	89.6 [2.6]			−2			0.87	4.8
		Site_A_	87.7 [3.8]			14			0.86	5.6
		Synth EchoNet	87.1 [3.7]			15			0.85	5.9
All	Camus (base)	Camus	93.3 [2.3]	95.7 [1.0]	92.0 [3.2]	1	0	1	0.84	2.6
	Site_A_		88.9 [3.4]	92.3 [2.1]	85.4 [5.3]	10	−2	14	0.85	4.8
	Synth Camus		81.2 [5.4]	90.3 [2.8]	79.6 [8.3]	33	7	−5	0.83	7.5
	Synth Site_A_	Site_B_	88.4 [3.8]	90.7 [3.4]	83.1 [9.4]	−9	−1	−11	0.83	5.4
	Site_A_ (base)		85.1 [4.0]	91.3 [2.0]^†^	84.4 [4.7]	−27	−8	8	0.84	7.5
	Camus		81.8 [4.9]	91.9 [1.8]^†^	86.6 [4.4]	−35	−10	−10	0.83	8.7
	Camus	Pathological	89.8 [2.6]	92.5 [1.8]	92.2 [2.0]	−9	−2	8	0.86	4.1
	Site_A_ (base)		89.0 [3.3]	92.0 [2.9]	87.5 [3.1]	−4	5	19	0.85	4.2
	Synth Site_A_		88.3 [4.9]	87.6 [4.1]	84.3 [6.1]	9	5	−2	0.84	4.6

LV_endo_ = left ventricle endocardium, LV_epi_ = left ventricle epicardium, LA = left atrium, A4C = apical four chamber, A2C = apical two chamber, ED = end diastole, ES = end systole. All refers to all annotations (LV_endo_, LV_epi_, and LA), views (A4C and A2C), and phases (ED and ES). $\boldsymbol {D}$ = Dice score, $\boldsymbol {MAD}$ = median absolute deviation, $\boldsymbol {B}$ = Bias percentage, $\boldsymbol {S_{p}}$ = simplicity, and $\boldsymbol {d_{m}}$ = mean average distance. For inter-observer $\boldsymbol {S_{p}}$ is listed for the second round annotations and $\boldsymbol {B}$ is calculated as $O_{2} - $Original.

†: Not statistically different with a P-value < 0.05.

### B. Learning From Synthetic Data

Networks were trained on Camus, EchoNet, Site_A_, and each of the synthetic datasets for the task of LV_endo_ segmentation in apical four chamber end diastole images. Networks were then tested on the EchoNet, Camus, Site_B_, and Pathological test sets. Results for EchoNet are shown in Table I and for the other three sets in Appendix D. On the EchoNet test set the networks trained on real EchoNet data unsurprisingly achieved the best results, but the network trained on synthetic data was comparable to both the networks trained on separate real datasets (Camus and Site_A_).

**Fig. 7.**
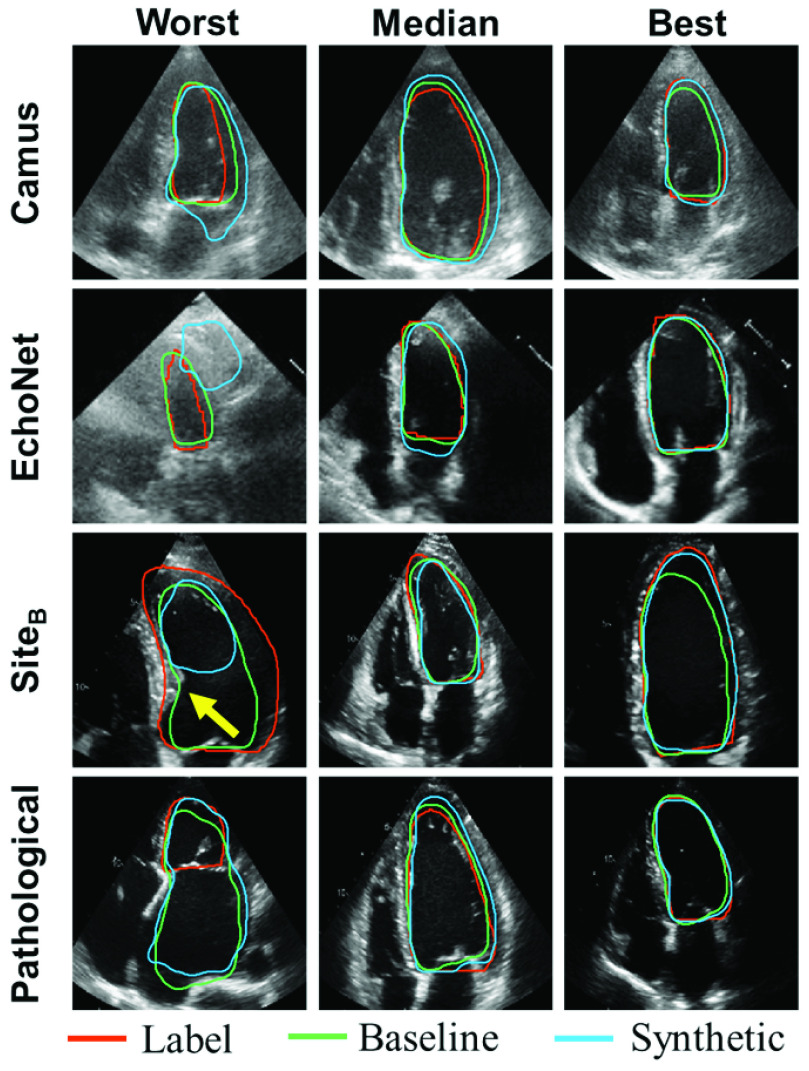
**Networks trained on synthetic data produce accurate segmentations in most cases:** Worst, median, and best LV_endo_ segmentation results on the Camus, EchoNet, Site_B_, and Pathological test sets for the network trained on the baseline real data and the synthetic data. The task for all networks was LV_endo_ segmentation in apical four chamber end diastole images. Images were ranked by Dice score for the network trained on synthetic data. The baseline and synthetic networks are always specific to the dataset (so for Camus the baseline network was trained on Camus and the synthetic network was trained on synthetic Camus). The yellow arrow points to a bulging septum in that image (see text). The baseline for Site_B_ and Pathological was Site_A_.

Qualitative results are shown in Fig. 7. In some cases the networks trained on synthetic data performed poorly. For example, in the worst case for Camus the network did not find the correct mitral valve cut-off plane. In the worst case for EchoNet the network found the wrong chamber, likely fooled by the strong reflective signal just beneath that resembles a valve. This image is also poor quality. In the worst case for Site_B_ the network misread the bulging septum (yellow arrow) as the mitral valve and cut off the segmentation there. The anatomical models were originally built from CT scans of asymptomatic patients and thus the segmentation network from synthetic images was not exposed to pathological cases (such as those with a bulging septum) during training. This was shown explicitly on the Pathological test set where the network failed to identify the LV given an enlarged LA (although the baseline network also failed in this case). However, these results were outliers. In most cases the network trained on synthetic data performed well with annotations that are similar to the manual labels and baseline.

Next, the robustness of the synthetic data was tested by extending the task to all annotations, phases, and views. We evaluated end diastole and end systole although the synthetic datasets do not contain end systole images. Results testing on the Camus, Site_B_, and Pathological test sets are shown in Table I. The network trained on the synthetic data performed worse in both cases on LA segmentation and for LV_endo_ segmentation in the Camus dataset. However, on Site_B_ the synthetic network outperformed all real datasets in LV_endo_ Dice and distance scores. There was a high positive Bias for the synthetically trained networks on Camus and a strong negative Bias for Site_A_ and Camus on Site_B_. The network trained on synthetic data was able to achieve similar performance to the networks trained on real datasets on the Pathological dataset for LV_endo_ segmentation, although LV_epi_ and LA Dice scores were slightly lower.

### C. Variability Analysis

To test the impact of parameters in the pipeline, synthetic datasets with tweaked parameters were generated and a segmentation network was trained for each. To test the effect of the transformation process, the pseudo dataset (before transformation with the CycleGAN) was compared to the synthetic dataset (after transformation with the CycleGAN). The Camus pseudo and synthetic datasets were compared to the EchoNet pseudo and synthetic dataset to analyze the effect of different parameters in the extraction process and different real datasets in the transformation process respectively. To test whether including additional variability helped, datasets extracted from just the 19 original anatomical models (Pseudo EchoNet* and Synth EchoNet*) were compared to datasets extracted from the set of 99 new shape models (Pseudo EchoNet and Synth EchoNet). To simplify results, all networks were trained for LV_endo_ segmentation only and were tested on the EchoNet test set since it was the largest.

Results are shown in Table II. Using the pseudo images provided a good baseline result even without the transformation process. Extending the anatomical model set as well as using dataset specific extraction processes slightly helped, but did not make a large difference. The transformation process did increase performance in the case that the correct dataset or a similar dataset was used (EchoNet/Site_A_). However, using the Camus dataset actually significantly degraded the results.

**TABLE II:** Evaluating Data Generation Variability: Median Results on the EchoNet Test Set for LV_endo_ Segmentation While Changing Various Parts of the Generation Pipeline. All Dice Results Except Pseudo Vs. Pseudo^*^ are Statistically Different With a p-Value < 0.05 (Computed With a Wilcoxon Signed-Rank Test). Results are Ordered by Dice Score and Bold Shows the Best Result

Train	Test	$\boldsymbol {D} (\%)$ [$\boldsymbol {MAD}$]	$\boldsymbol {B}$ (%)	$\boldsymbol {d_{m}}$
Synth EchoNet	EchoNet	87.1 [3.7]	15	5.9
Synth Site_A_		86.8 [4.0]	14	6.2
Synth EchoNet^*^		86.5 [4.0]	17	6.1
Pseudo EchoNet^*^		84.4 [5.3]^†^	14	6.7
Pseudo EchoNet		84.1 [4.5]^†^	18	6.7
Pseudo Camus		83.3 [4.7]	9	7.0
Synth Camus		81.9 [6.9]	13	7.3

^*^: these datasets were extracted from only the original 19 anatomical models rather than the extended set including the new shape models from PCA.

^*^: Not statistically different with a P-value < 0.05.

## V. Discussion

We developed a fully automated^4^ pipeline for generating large annotated datasets for training CNNs from anatomical models. The generated synthetic images look realistic and expert annotations on the synthetic images closely matched those from the pipeline. Moreover, segmentation networks trained from the synthetic datasets produced accurate segmentations on real images in most cases. Dice scores from the synthetically trained networks were comparable to inter-observer errors and networks trained on a separate set of real data.^4^Other than the manual step of selecting the CycleGAN epoch, which does not significantly impact results.

### A. Generated Images and Annotations

**Fig. 8.**
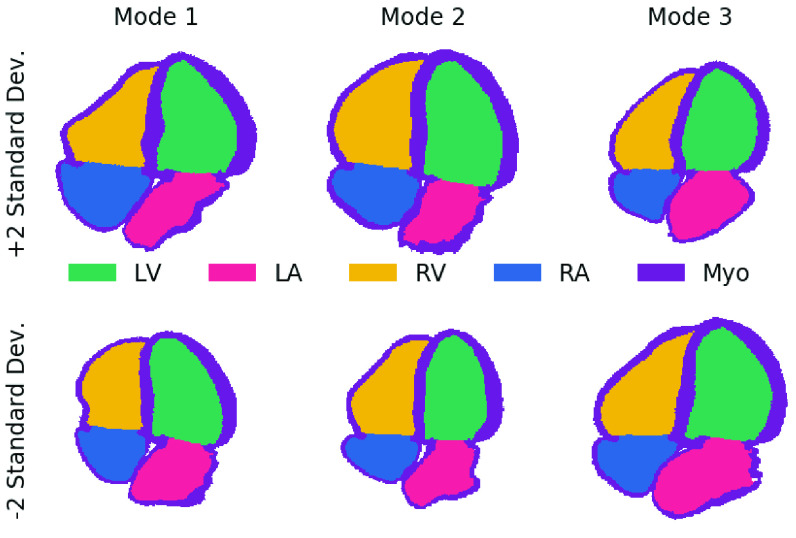
**Shape variations are mainly seen in the LA, not the LV:** Four chamber slices showing ± 2 standard deviations from the mean model for the first three modes calculated using principal component analysis (see Sec. II-A.1). LV = left ventricle, LA = left atrium, RV = right ventricle, RA = right atrium, and Myo = myocardium.

We found that the expert annotations on synthetic images closely matched the ones generated by the pipeline. This indicates the paired synthetic images and labels are accurately delineating the LV in a manner consistent with expert expectations. Dice scores between experts and the anatomical model were lower (although still comparable) for the LA. To explain this, Fig. 8 shows samples from the first several modes of the shape analysis described in Sec. II-A.1. The anatomical models show complex LA shapes as well high variability in shapes between different models. However, the LA is typically still annotated as a half-ellipsoid shape by the annotators (similar to the LV - see Fig. 3) in images and we hypothesize the lower scores were due to this difference in annotation complexity. Apical images are typically optimized for image quality in the LV rather than the LA, which may hinder accurate labeling of detailed LA shapes.

The inter-observer LV_endo_ Dice scores for real images presented here are lower than those presented by Leclerc *et al.* on the same tasks [30]. There are two likely contributors to this. First, as discussed in Sec. III-C, a lack of explicit guidelines can cause differences in standard practice at different clinics and our results measure experts practicing in different sites. Second, in our inter-observer study the annotators were only given access to a single frame during the second round. This was necessary since the current pipeline only generates a single frame, but the lack of myocardial movement inhibits accurate detection of the compacted myocardium and other features. While more difficult, it also matches the task of the segmentation network, which is given a single frame only, and thus represents a better comparison for the pipeline.

### B. Learning From Synthetic Data

We evaluated segmentation networks trained from synthetic data. First, we tested LV_endo_ segmentation in apical four chamber end diastole images and then extended the task to LV_epi_ and LA segmentation in apical four chamber and two chamber views and end diastole and end systole phases. Since there are numerous examples of deep learning methods failing once deployed due to implicit bias in the training dataset, we extensively validated our approach using five different datasets from various institutions and annotators. All hyperparameter tuning and initial tests were conducted using only a single dataset (Camus) and we then tested the same pipeline on additional unseen datasets. In some cases implicit bias towards the Camus dataset in the pseudo generation step were observed (see Appendix G), but the pipeline is still able to adapt and produce good results across datasets. This robustness is a strength of our work.

The network was able to achieve comparable results to a network trained on a separate real dataset. In a review of the results, failure cases primarily occurred when the network struggled to properly identify the mitral valve plane in real images (such as the worst case in Site_B_ of Fig. 7). Since the valve is included in the anatomical models as a flat disk, the synthetic images do not contain the same variation of valve appearances of real datasets. Including a variety of valve structures in the synthetic images is one way the proposed pipeline could be improved. The network trained on synthetic data was generally able to segment images from the Pathological dataset well, but could not properly identify the LV in cases with an enlarged LA (shown in Fig. 7 and in supplementary material). However, networks trained on real data also struggled on these images indicating that these cases would likely require expert review and adjustments regardless of the dataset used. If a known pathology should be handled, the models could also be adjusted to include this by including a single anatomical model exhibiting this pathology and using the PCA shape analysis to generate variations compared to a healthy normal model.

### C. Clinical Applicability

LV_endo_ segmentation is used clinically for an estimation of volumes and ejection fraction which are important measures of the efficiency of heart function. Clinical measures are not presented here because metric pixel sizes are not given for the datasets. However, previous studies have shown a strong correlation between the accuracy of Dice scores and the accuracy of predictions of clinical parameters across multiple algorithms and inter-/intra-observer studies (correlation coefficient of −0.92 between Dice scores at end-diastole/end-systole and ejection fraction mean average error across 12 experiments) [30]. Thus, the small decrease in accuracy of Dice scores presented here would likely result in a small decrease in accuracy of clinical metrics. The Dice scores obtained with the synthetically trained networks are still within the range of inter-observer error, indicating the same would likely be true for clinical metrics. Annotators rely on visually tracking the same point across the cycle to ensure consistency between predictions at end-diastole and end-systole and ejection fraction prediction could also be improved by including this temporal coherence between the predictions at different phases in the segmentation networks (using recursive neural networks for example).

### D. Variability Analysis

We also analyzed potential sources of error for the networks. When testing images trained on one dataset on a different dataset there are two primary elements that cause decreased performance:

1) **Texture differences:** In echo these are linked to acquisition changes such as varying ultrasound machines, gain, focus, resolution, and other imaging parameters. In the proposed pipeline, texture primarily comes from the transformation step.
2) **Shape differences:** Due to a) differences in the width/depth of the acquisition which change tissue shape in the produced image, b) changes in the underlying tissue shape, or c) differences in annotation style. In the proposed pipeline, shape changes comes from the extraction step. Annotation style is linked to the original anatomical models.

Texture and shape differences were previously explored in object recognition where Geirhos *et al.*, who showed that CNNs trained on ImageNet for classification were more biased towards changes in texture than shape [37]. We tested these differences in echo segmentation in Table II, using the generation pipeline to isolate the impact of each component.

Changes in shape due to imaging parameters were isolated by varying the width/depth/percentage of LV focused images in the two pseudo datasets and had a very small effect. Changes in underlying tissue shape were isolated by comparing the datasets built from the original models (Pseudo EchoNet*/Synth EchoNet*) to the set of models containing additional variability from the shape extension in Sec. II-A.1 (Pseudo EchoNet/Synth EchoNet). Changes in results were small and reversed between the pseudo and synthetic sets. This is likely because there were minimal variations in LV shape. As shown in Fig. 8, the largest changes in the LV are variations in size and width. Modifications to these parameters are already included in the pseudo image generation process, thus the shape extension did not add significant new variations of LV shape to the dataset. Pathological changes in the underlying shape (such as the bulging septum or enlarged left atrium in Fig. 7) do seem to reduce segmentation accuracy. To include these elements in the pipeline, new models could be built from pathological cases as discussed above.

Texture changes were isolated by comparing different synthetic datasets using CycleGANs tuned to different real datasets since the same underlying shape was used in all cases. Results showed that image appearance could make a significant difference as the Synth Camus network performed significantly worse than Synth EchoNet/Synth Site_A_. This matches the qualitative appearance difference between EchoNet/Site_A_ and Camus in Fig. 7. Results here also showed that solid performance could be obtained with only the pseudo network. This is an encouraging result indicating that applications without high accuracy needs could further simplify the pipeline by removing the transformation step.

**Fig. 9.**
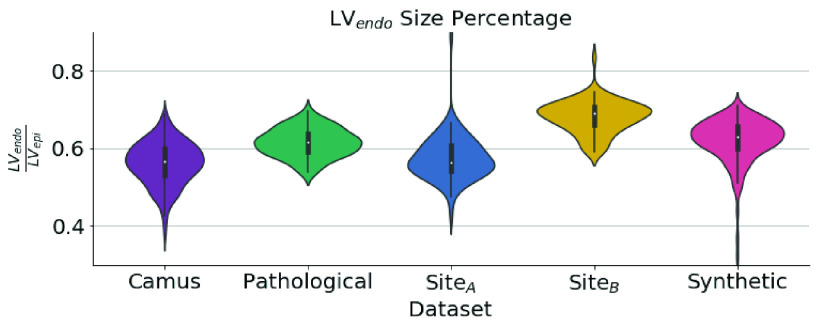
**Annotation bias can yield large differences:** Violin plot showing the amount of LV_epi_ area comprised of the LV_endo_ area for the labels in each dataset. A lower value indicates a thicker myocardium. EchoNet does not have LV_epi_ labels.

Assuming that human observers are adept at adapting to differences in texture and shape, differences in annotator style can be isolated from the inter-observer study presented in Table I. Differences between observers were substantial both in terms of Dice score and Bias, indicating a systematic difference between annotators. Although there were various constraints in this study (as discussed above), this difference was also clearly present in the original datasets without those constraints. Fig. 9 shows the ratio of LV_endo_ area to LV_epi_ area for the labels of each dataset which generally corresponds to the thickness of the labeled myocardium. This percentage is much higher (indicating a thinner myocardium) for the synthetic datasets than all the other datasets excluding Site_B_. While this difference could instead indicate the prevalence of pathologies (e.g. hypertension) in the dataset, we present additional validation in Appendix E that the differences in Fig. 9 are primarily due to changes in annotation style. Results also match previous studies showing echo measurements typically overestimate the thickness of the myocardium [38]–[40].

Our segmentation results also point towards annotation style as the critical factor in determining accuracy. Bias was high for LV_endo_ results for networks trained on synthetic data on all other datasets than Site_B_. On the other hand, networks trained on real datasets had a high negative Bias when tested on Site_B_. The increase in Bias was correlated with lower Dice sores and higher mean distances, but not with simplicity, showing that the segmentations were still well-formed. This Bias was not observed for LV_epi_ in Table I indicating that the variation comes purely from the differences in LV_endo_ annotation style. The high performance of the synthetic network on Site_B_ matches both Fig. 9 and the low bias with $O_{2}$ in the inter-observer study since $O_{2}$ labeled Site_B_.

Therefore, the primary reason for decreased performance in our experiments (for networks trained on both synthetic and real data) was differences in annotation style, with texture differences playing a secondary role. Other than several outlier cases, the networks trained on synthetic data performed well and produced well-formed segmentations. One of the advantages of the pipeline proposed in this work is that the same annotation style can be applied to images from any dataset which will bring consistent performance for a network implemented in clinical practice. Given that the synthetic images are built from anatomical models derived from CT images, the synthetic images generated can be used to standardize annotation style.

### E. Extensions and Future Applications

An abundance of augmentation techniques exist specifically for improving segmentation performance on limited datasets. For example, several authors introduced method based on statistical models to modify images following the deconstructed natural shape variation [8], [9]. Methods such as Jafari *et al.* [41] or Shin *et al.* [42] use GANs to expand the dataset with new natural images. This work focuses on the performance of the standard pipeline rather than one with augmentations tuned for a specific application, but these techniques, as well as any other task-specific augmentation techniques (or loss functions), could readily be applied here to improve results.

While we implemented the pipeline for 2D LV/LA echo segmentation to enable comparison against existing techniques, one of the strengths of our method is that the anatomical models are 3D and contain annotations for a variety of tissue types. Moreover, our method is not limited to ultrasound and a paired database of CT or MRI images could also be generated using this method. The pipeline is theoretically extensible to any segmentation or landmark detection task. Extension requires a) a small set of anatomical shape models similar to those described in Section III-A, b) a real dataset of unlabeled images from the relevant modality and view, and c) code to extract a slice from the anatomical models matching real images. Part c) can be accomplished through an analysis of important landmarks present in the relevant images that are also defined in the model. Additional unforeseen challenges likely exist for adapting to new anatomies and modalities, but we anticipate the ability to overcome these.

In addition to testing the pipeline on novel applications, future work will focus on adapting the pipeline to 3D, which is increasingly being used in clinical practice, but where manual labeling is even more difficult. The difficulty of manual labeling has thus far limited the development of benchmark datasets which is why the focus of this validation work is limited to 2D images. While challenging, other groups have previously shown the ability to adapt generative networks for 3D medical image synthesis (for example [42] and [43]). Due to GPU memory constraints these works required use of lower resolution volumes, a challenge for adapting the existing pipeline as well. The anatomical models could also be used as context for generation and/or segmentation as was proposed in [44]. Additionally, one of the strengths of echo is the high temporal resolution. Future work will also focus on extending image generation techniques to include labels and images across the cardiac cycle.

## VI. Conclusion

Building large annotated datasets can be difficult and time-consuming. For cases where a small percentage of outliers are acceptable, or a confidence metric can be designed to catch outliers, we present a method to train a cardiac segmentation network with zero manual labeling required. The generated labels represent an accurate ground truth, can be rapidly built, and grant additional flexibility since the anatomical models providing the ground truth can be automatically adjusted as required. By eliminating or reducing labeling requirements, the proposed pipeline enables greatly accelerated deep learning algorithm development in cardiac imaging.
